# A full Brazilian or all natural: understanding the influences on young women’s decision to remove their pubic hair

**DOI:** 10.1186/s12905-019-0868-1

**Published:** 2019-12-19

**Authors:** Patricia Obst, Katherine White, Ebony Matthews

**Affiliations:** School of Psychology and Counselling, Queensland University of Technology, Victoria Park Road. Kelvin Grove, Qld, Brisbane, Australia

**Keywords:** Pubic hair removal, Theory of planned behaviour, Young women

## Abstract

**Background:**

Research indicates that young women are being exposed to increasing pressures to remove pubic hair from their bodies, which has the potential for both negative physical and psychological consequences. Women’s personal choice and reasoning for partaking in pubic hair removal is influenced by broader social influences; however, there is little theory-based research drawing from established decision-making models investigating the underlying processes that lead young women to engage in pubic hair removal practices. Based on the Theory of Planned Behaviour, it was hypothesised that 1) attitude, subjective norm, and perceived behavioural control would predict intention to remove pubic hair; 2) additional variables (prototype similarity and favourability) from the Prototype Willingness Model would significantly predict intention to remove pubic hair; 3) feminist values would significantly predict decreased intention to remove pubic hair; and 4) intention and perceived behavioural control would predict future self-reported removal of pubic hair.

**Method:**

The current study included a sample of 270 young women (17–25 years old), who completed an online survey and a follow up survey 4 weeks later (*N* = 96).

**Results:**

Attitudes, perceived behavioural control, and similarity to prototypical pubic hair removers were significant predictors of intention to remove pubic hair. Intention was significantly positively associated and feminist values were significantly negatively associated with actual pubic hair removal.

**Conclusions:**

These findings align with Theory of Planned Behaviour propositions. Furthermore, the expansion of the model highlights how broader social images impact on young women when deciding whether to engage in a behaviour that is intimately associated with their body image.

## Background

Hair removal in varying forms has been practiced across many cultures for centuries [1]. In the developed world, there has been a recent rise in more extreme forms of hair removal and a burgeoning of a whole industry offering hair removal technologies, such as laser hair removal and electrolysis, which can result in permanent hair removal. Research indicates that young women are being exposed to increasing pressures to remove pubic hair from their bodies, which has the potential for both negative physical and psychological consequences [2]. To date, there is little theory-based research drawing from established decision-making models that investigate the underlying processes that lead young women to engage in hair removal practices. Results of studies that have examined modern hair removal practices in large female samples indicate that the large majority of participants (over 90%) report having removed pubic hair (e.g., 3, 4, 5). There are some differences in prevalence, with Caucasian women being more likely to remove pubic hair than women of other ethnicities and a higher prevalence of removal in younger women [3, 4].

Over half of the young women surveyed across studies experienced at least one physical complication due to pubic hair removal [5–7]. The most common side effects found were epidermal abrasions, infected ingrown hair, severe itching rashes, and other skin infections such as molluscum contagiosum. Complications specifically associated with laser hair removal include blistering, hypo/hyper-pigmentation, scarring, textual changes, uveitis, iritis, and infections such as herpes simplex and bacterial infections [8]. When exposed to societal expectations regarding body image and hairlessness, young women may also experience psychological harm. In Kitzinger and Willmott’s [9] study of women with an increased growth of body hair due to Polycystic Ovarian Syndrome, participants reported feeling ‘freakish’ and ‘unfeminine’ due to their hair growth. They referred to their hair as “unsightly, distasteful, upsetting, embarrassing, and dirty” (10, page 353). Qualitative studies by Fahs [10, 11] indicate that, while the women in their studies stated that body hair removal was their ‘choice’, they expressed disgust towards women who did not remove body hair labelling them as ‘unclean’, ‘dirty’, ‘gross’, and even ‘unnatural’. In Fah’s second study [10], participants were required to not remove body hair for 10 weeks. Participants reported feeling dirty, sexually unattractive, and disgusting, admitting that other people had expressed disgust at their body hair. Such findings indicate how strongly internalised this social norm is for many women and the strong negative feelings associated with their natural body hair.

The idea of hair being dirty and hair removal being undertaken for reasons of cleanliness is a common finding in the research. For example, in a sample of Australian women, Tiggemann and Hodgson [12] found the most cited reason for removing pubic hair was “it makes me feel cleaner” followed by “sexual attractiveness” (page 892). In a study by Riddell, Varto, and Hodgson [13], the top four reasons for removing pubic hair after wearing a bathing suit, were feeling “more comfortable”, “feminine”, “clean and hygienic”, and “sexual attractiveness” (page 125). Such findings are repeatedly supported across studies with pubic hair removal reported as feminine, clean, and attractive while pubic hair is considered unattractive, unclean, and gross [6, 14, 15].

Braun, Ticklebank, and Clarke [2] argue that the choice to remove or not to remove hair has become constrained by the alternative meanings coexisting around hair removal. Ideas of cleanliness, attractiveness, femininity, and sexuality surrounding the removal of hair have diminished the degree to which hair removal can be accepted as an unencumbered choice. These coexisting meanings have been evolving slowly across generations. A seminal study by Hope [16], which examined advertisements in Harper’s Bazar, found body hair has long been targeted in advertisements, beginning with underarm hair back in 1915, moving to leg hair in the 1940s, with current beauty and fashion media now promoting pubic hair removal [17].

With such strong messaging about female body hair, it is unsurprising that one of the common reasons given by women for removal of hair is to feel more feminine [6]. However, as far back as 1968, Women’s Liberationists have questioned the association between body hair, masculinity, femininity, and feminism [18]. Such associations have added another layer of meaning to body hair, as actively not removing body hair is often viewed as a symbol of feminism [15, 16, 19–21]. However, little research has explored the relationship between feminist ideology and women’s decisions to engage in pubic hair removal, particularly within a theory based decision-making format.

While research has been conducted on the motivations and beliefs surrounding pubic hair removal, to date no theoretical models of decision-making have been applied to further understand young women’s engagement in this behaviour. The Theory of Planned Behaviour [22] is a well validated and extensively used model of decision making that may aid in the understanding of young women’s decision to engage in extensive hair removal practices. In the Theory of Planned Behaviour framework, intention is the most proximal determinant of people’s behaviours. The three determinants of intention are attitudes (how favourably/unfavourably people feel about performing the behaviour), subjective norm (perceived pressure from others to perform or not perform the behaviour), and perceived behavioural control (perceptions of control over performing the behaviour, also believed to have a direct impact on behaviour). Underlying these three determinants are behavioural beliefs (advantages/disadvantages of performing the behaviour); normative beliefs (referents who would approve/disapprove of performing the behaviour); and control beliefs (specific barriers/facilitators of behavioural performance). The belief component of the Theory of Planned Behaviour provides an additional aspect of practical application to the model as the identification of important beliefs can then inform the development of empirically-based strategies to challenge people’s perceptions, often leading to a change in behaviour [23].

The Theory of Planned Behaviour framework, with an ability to explain on average 27 to 39% of variation in intention and behaviour, respectively [24], has been applied to a wide range of behaviours. However, the Theory of Planned Behaviour has been criticised for its parsimony; with the standard constructs potentially only partially explaining the variance in behaviour [25]. Given the strong influence of the media and the strong images associated with women who do or do not remove pubic hair reported in the research, constructs from the Prototype Willingness Model [25] may be useful to include as an extension to the Theory of Planned Behaviour constructs in understanding hair removal behaviours. The Prototype Willingness Model is comprised of two different pathways (reasoned and reactive) that attempt to explain decision-making [26–29]. The reactive pathway of the Prototype Willingness Model is more image driven, and less intentional. A key construct of reactive decision making is that of prototypes. Prototypes are social images of a person who does and does not engage in the specific behaviour. As noted, images and messaging associated with hair removal are widely used in the media, thus, the inclusion of prototypes may be particularly relevant in the context of women’s hair removal. The Prototype Willingness Model examines both prototype favourability (how positive participants feel about prototypical images of someone who removes or does not remove their pubic hair) and prototype similarity (how similar participants feel to those prototypes).

### Current research

Previous research indicates that women’s personal choice and subjective reasoning for partaking in pubic hair removal may be influenced by values, such as feminist ideologies and broader social influences, as the social norm of pubic hair removal is becoming a deeply embedded part of Western culture. Research that focuses on the underlying decision-making processes that lead young women to remove pubic hair can aid in the development of theoretical and evidence-based strategies to provide young women with the information they need to make informed choices about their hair removal decisions. The current study applies an extended Theory of Planned Behaviour framework that includes the Prototype Willingness Model constructs of prototype similarity and prototype favourability as well as a measure of participants’ feminist values to the pubic hair removal behaviour of young Australian women aged between 17 and 25 years old. This research is the first study to systematically explore pubic hair removal behaviour through the application of an established theoretical model of decision-making.

According to the theoretical premises of the Theory of Planned Behaviour it was hypothesised that:

(H1) Attitude, subjective norm, and perceived behavioural control would predict intention to engage in pubic hair removal;

(H2) The additional variables from the Prototype Willingness Model of prototype favourability and prototype similarity, both for pubic hair removers and non-removers, would add significantly (positively and negatively respectively) to the prediction of intention to remove pubic hair;

(H3) Endorsement of feminist values would further add to the prediction of intention to engage in pubic hair removal behaviours (with a negative association);

(H4) Intention to engage in pubic hair removal and perceived behavioural control would significantly predict subsequent self-reported pubic hair removal behaviour, four weeks later.

## Methods

### Participants

Study participants comprised a convenience sample of young adult women aged 17 to 25 years residing in Australia. Fifteen young women participated in the pilot study and 270 in the main study, 96 of whom responded to the follow-up survey. The majority of the sample were Caucasian (94.7%) and heterosexual (75.7%), while 20.0% identified as bisexual and 3% as homosexual. Over half of participants were in a committed relationship (53.9%), while the rest were single or casually dating. Nearly half were casually employed (42.6%), 24.0% were employed full-time, 18.0% were employed part-time, and 11.0% were unemployed. A majority of the sample was educated, with 65.8% holding a Bachelor’s degree or higher (61.6%), 10.3% completed a Diploma or training certificate, and 20.2% held a high school certificate. Almost half the sample earned less than $20,000 a year (46.4%), with 36.5% reporting earnings of $20,000 to $50,000 a year and 16.8% earning above $50,000 a year.

### Procedure

The research consisted of an online pilot study, and a two-part online survey that assessed pubic hair removal behaviour in young women, approved by the Queensland University of Technology’s Human Research Ethics Committee (1700000408). Participants in the pilot study were undergraduate students approached via email. Participants for the main and follow-up surveys included young adult women who were recruited through an all-women’s Facebook group called ‘Girls Advice’, as well as posts to other female oriented Facebook pages. The material gathered from the pilot study assisted in the creation of the main survey (see measure section). The open online surveys were administered through the online platform Key Survey. Participants provided their consent by clicking on next after reading the participant information provided before entering the online survey. The main survey was open for 3 months. On completion of the main survey, participants were invited to provide contact details to participate in the follow-up survey. The follow-up survey was administered four weeks after the main survey and assessed hair removal behaviour over the previous month. Participation in both of the surveys was completely voluntary, with no forced response required, but respondents were not able to review answers once an item was completed. Participants were awarded university course credit or the opportunity to enter into a prize draw. IP addresses for participants were not collected, but data was screened for duplicate entries base on unique identifier codes. Response rates based on the number of participants who completed the survey as a proportion of those who clicked on the links were 90.5% for the pilot study, 39.2% for the main study, and 88.2% for the follow up study. Please see additional material for full copies of all surveys.

### Measures

#### Pilot questionnaire (24 items 4 pages, see Additional file 1)

##### Traits

To inform the prototype aspects of the main study so as to understand the images women hold regarding pubic hair removal, participants were asked to describe the typical woman who does and does not remove pubic hair. Results indicated that participants viewed a woman who removed their pubic hair as normal, clean, and adventurous, whereas they believed a woman that did not remove their pubic hair was self-confident, unbothered by others opinions, and alternative/independent thinker, conservative, and reserved. The most frequently cited of these traits were included in the main questionnaire for descriptive analysis.

##### Indirect beliefs

To elicit the key beliefs underlying participants’ attitudes, subjective norms, and perceived behavioural control regarding pubic hair removal, they were also asked to report what they felt were the main advantages/disadvantages of pubic hair removal (behavioural beliefs), who would or would not endorse them engaging in this behaviour (normative beliefs) and what were the key facilitators and barriers (control beliefs). Content analysis of the qualitative transcripts was used to elicit the most frequently endorsed key beliefs for inclusion in the main questionnaire.

#### Main questionnaire (75 items, 9 pages, see Additional file 1)

Participants completed 55 Theory of Planned Behaviour and Prototype Willingness Model items. Item were developed based on the established protocols for assessing constructs in these models [22, 26]. For these items, the target behaviour assessing pubic hair removal was worded (with an initial explanation/example provided) in relation to the removal of ‘privately visible hair’ to distinguish it from other items as part of a broader survey, which also examined a variety of other hair removal practices in body areas easily visible. Theory of Planned Behaviour and Prototype Willingness Model items were worded in relation to the next 4 weeks in accordance with the Theory of Planned Behaviour’s guidelines and had a seven-point Likert style response format unless specified otherwise. A small number of items were negatively worded to avoid response sets; these items were reverse scored prior to data analysis.

##### Indirect beliefs

Endorsement of the Indirect beliefs derived from the pilot study was assessed through participant responses to a series of questions asking the likelihood (*1 - very unlikely to 7 - very likely*) (i) that their pubic hair removal would result in the following: feel attractive, feel clean, be socially acceptable, feel comfortable, feel confident, reduce negative judgments from others, be time consuming, be effortful, be expensive, be painful (behavioural beliefs); (ii) that the following groups/people would think that participants should remove their pubic hair: friends, partner, family, media, beauty industry, sex industry, feminist groups, alternative people/groups, and society (normative beliefs); and (iii) that the following factors would prevent participants from engaging in pubic hair removal: lack of time, lack of equipment, and laziness (control beliefs).

##### Attitude

Participants’ attitudes towards performing pubic hair removal was measured using four semantic-differential scales (e.g., (*1-favourable to 7-unfavourable*) (α = .89).

##### Subjective norm

Perceived pressure from others to engage in pubic hair removal was assessed with three statements (e.g., Most people who are important to me would approve of my engaging in the removal of hair that is privately visible in the next four weeks.) that participants responded to on 7-point scale (*1-strongly disagree to 7-strongly agree*) (α = .72).

##### Perceived behavioural control

Perceived behavioural control was assessed by asking participants to rate how strongly they agreed (*1-strongly disagree to 7-strongly agree*) with the statement “I am confident that I can engage in the removal of hair that is privately visible in the next four weeks.”

##### Prototype favourability

Participants were asked to rate how favourably (*1-very unfavourable to 7-very favourable*) they felt towards a typical woman who removes/does not remove her pubic hair.

##### Prototype similarity

Prototype similarity was assessed by asking participants how similar (*1- very dissimilar to 7-very similar*) they felt to a typical woman who removes/does not remove her pubic hair.

##### Behavioural intention

Behavioural intention to remove pubic hair was assessed by using the mean total of two items regarding the likelihood the participant would remove their hair in the next four weeks, and if they intended to remove their hair in the next four weeks (*1-very unlikely to 7-very likely*) (*r* (268) = .93, *p* < .001).

##### Feminist values

Feminist values were measured using a short 10 item version of the Feminist Ideology Scale (FIS) by Morgan [30] that assesses participants’ thoughts and feelings towards historical and current gender discrimination and subordination. Participants were required to respond to their agreement (*1-strongly disagree to 6-strongly agree*) with 10 statements (e.g., ‘Women have been treated unfairly on the basis of their gender throughout most of human history’) (*α* = .90).

#### Follow-up questionnaire (15 items, 2 pages, see Additional file 1)

##### Hair removal behaviour

Actual pubic hair removal behaviour was assessed in a follow-up survey, four weeks after the main study was completed. Two items asked participants how often they had removed their pubic hair in the previous four weeks (1-never to 7–very often) and to what extent (*1-not at all to 7-a great extent*) (*r* (94) = .704, *p* < .001).

## Results

IBM Statistical Package for the Social Sciences, SPSS version 23 was used to conduct all statistical analyses. Missing values analysis revealed data was missing completely at random, *p* = .041 and list wise deletion was employed. Assumptions related to hierarchical regressions were assessed and the data were deemed appropriate to conduct the analyses.

### Descriptive statistics

Ninety-eight percent of the women who responded to the main survey reported that they had engaged in pubic hair removal of some kind in the past. Shaving was the most common method of removal reported (75.7%), followed by laser/electrolysis removal (9.6%) waxing (9.2%), and removal creams (1.6%). When asked to report the current status of their pubic hair, 52.3% responded they had a full Brazilian (the removal of all pubic hair from the pelvic region, vulva, labia, perineum, and anus, sometimes leaving a thin strip of hair on the mons pubis); 9.8% a French/playboy style (removing all the hair from the panty line, the top and the hair in the inner labia), 19.5% had just removed the hair from their bikini line, and 18.3% reported they were currently all natural.

Endorsement of prototype traits of pubic hair removers and non-removers revealed that participants rated pubic hair removers (*M* = 5.15, *SD* = 1.08) as significantly more “normal” compared to non-pubic hair removers (*M* = 4.75, *SD* = 1.12; *t* (261) = 5.39, *p* < .001). In terms of traits specifically describing removers and non-removers, women who removed their pubic hair were more highly endorsed as “clean” (*M* = 5.70, *SD* = 1.1) than adventurous (*M* = 4.53, *SD* = 1.14) or self-conscious (*M* = 3.34, *SD* = 1.33; *F* (2, 266) = 119.33, *p* < .001), while women who did not remove their pubic hair were more highly rated as self-confident (*M* = 5.14, *SD* = 1.67), than conservative (*M* = 3.86, *SD* = 1.17) or a feral/hippy (*M* = 3.53, *SD* = 1.23; *F* (2, 258) = 139.51, *p* < .001).

### Model testing

As shown in Table 1, all variables were correlated with both intention to engage in pubic hair removal and pubic hair removal behaviour. Attitude, perceived behavioural control, and similarity to pubic hair removers showed the highest correlations with intention to remove pubic hair and intention had the highest correlation with behaviour.

**Table 1:** Mean, Standard Deviations and Bivariate Correlations of all Variables in Regression Model

Variable	M	SD	1	2	3	4	5	6	7	8	9	10
1.Behaviour	3.71	1.62	–									
2.Intention	5.43	1.71	.69**	–								
3.Attitude	4.60	1.36	.58**	.58**	–							
4.Subjective norm	4.13	1.33	.25*	.21**	.30**	–						
5.PBC	5.88	1.42	.40**	.61**	.45**	.19**	–					
6.Favourability remover	4.65	1.10	.22*	.29**	.40**	.27**	.21**	–				
7.Favourability non- remover	4.30	1.06	−.27**	−.21**	−.27**	−.20**	−.13*	−.01	–			
8.Similarity to remover	4.84	1.26	.55**	.62**	.56**	.28**	.46**	.52**	−.22**	–		
9.Similarity to non-remover	4.19	1.40	−.52**	−.46**	−.48**	−.28**	−.29**	−.27**	.53**	−.49**	–	
10.Feminist values	43.6	8.40	−.33**	−.25**	−.26**	−.06	−.19**	−.10	.18**	−.16**	.16*	–

Note. * *p* < .05. ** *p* < .01 ***p < .001

#### Predictors of intention and behaviour to remove pubic hair

The model predicting intention to engage in pubic hair removal in the next four weeks was tested via hierarchical regression. Attitude, subjective norm, and perceived behavioural control were entered in the first step; the variables of prototype favourability of removers and non-removers, prototype similarity of removers and non-removers, and feminist values were added in the second step of the model.

As can be seen in Table 2, the standard Theory of Planned Behaviour constructs (Model 1) explained a significant 48.0% of the variance in intention, *F* (3, 238) = 72.85, *p* < .001. However, subjective norm was not a significant unique predictor of intention in this step. The constructs entered at step 2 explained an additional 10.0% of the variance in intention*, ∆F* (5, 230) = 10.92, *p* < .001. Attitude and perceived behavioural control remained significant unique predictors in the final model, with both similarity to prototypical women who remove and do not remove their pubic hair significant unique predictors of intention. Neither prototype favourability measures nor feminist values emerged as significant predictors of intention.

**Table 2:** Hierarchical Regression of Extended TPB Model on Intention to Remove Pubic Hair

	Variable	Β [95% CI]	β	∆R^2^	sr^2^
Model 1	Attitude	.49 [.36, .62]	.39***	.48***	.18
	Subjective norm	.02 [−.11, .14]	.01		.00
	PBC	.52 [.40, .64]	.43***		.22
Model 2	Attitude	.25 [.11, .39]	.20**	.10***	.05
	Subjective norm	−.05 [−.17, .06]	−.04		.00
	PBC	.40 [.28, .52]	.33***		.16
	Favourability remover	−.11 [−.27, .05]	−.07		.01
	Favourability non remover	.09 [−.07, .26]	.06		.01
	Similarity remover	.46 [.29, .62]	.34***		.12
	Similarity non remover	−.21 [−.36, −.07]	−.17**		.03
	Feminist values	−.01 [−.03, .00]	−.07		.01

Note. Β = unstandardized coefficients; β = standardised coefficients; sr^2^ = squared semi-partial correlations. ** *p* < .01. *** *p* < .001

Eighty one percent of the follow up sample reported having removed their pubic hair to some extent in the last four weeks. To test the prediction of follow-up behaviour (self-reported pubic hair removal in the last four weeks), a bootstrapped hierarchical regression was performed due to the smaller sample size (*n* = 96). Intention to remove pubic (only privately visible) hair and perceived behavioural control were entered as the first step and all other variables (attitude, subjective norm, prototype favourability of removers, and non-removers, prototype similarity of removers and non-removers, and feminist values) were added in the second step.

As seen in Table 3, Model 1 explained nearly half of the variance (*R*^*2*^ = 43%, *F* (1, 93) = 75.16, *p* < .001) in pubic hair removal, with intention the only significant unique predictor of behaviour. When the other variables were added in step 2, they accounted for a significant additional 8% (*∆F* (6, 86) = 2.46, *p* = 03) of variance in behaviour. Intention remained a significant unique predictor and feminist values emerged as a significant negative predictor of behaviour *(β* = −.22, *p* = .008).

**Table 3:** Bootstrapped Hierarchical Regression of Extended TPB Model on Pubic Hair Removal Behaviour

	Variable	Β BCA [95% CI]	p	β	∆R^2^	sr^2^
Model 1	Intention	1.57 [1.27,1.83]	.001	.70***	.43***	.40
	PBC	.00 [−.39, .49]	.997	.04		.00
Model 2	Intention	.91 [.48,1.29]	.001	.41	.08*	.13
	PBC	−.19 [−.73,.30]	.408	−.06		.01
	Attitude	.62 [.06,1.29]	.057	.20		.05
	Subjective norm	.23 [−.37,.87]	.445	.07		.01
	Favourability remover	.10 [−1.14,.71]	.847	.02		.00
	Favourability non remover	−.03 [−.82,.69]	.947	−.01		.00
	Similarity remover	.36 [−.20,.96]	.204	.11		.01
	Similarity non remover	−.40 [−.86,.25]	.141	−.14		.02
	Feminist values	−.10 [−.19,-.01]	.008	−.22**		.07

Note. Β = bootstrapped unstandardized coefficients; β = standardised coefficients; sr^2^ = squared semi-partial correlations. Bootstrapped * *p* < .05. ** *p* < .01. *** *p* < .001

### Critical beliefs underlying intention to removal pubic hair

Indirect belief means, standard deviations, and correlations are shown in Table 4. To identify the critical beliefs associated with young women’s intention to remove their pubic hair, analyses followed the guidelines by von Haeften, Fishbein, Kasprzyk, and Montano (2001; 24). Pearson product-moment correlations were first analysed to identify beliefs that significantly correlated with intention. Significantly correlated beliefs were then entered into a stepwise multiple regression as predictors of intention. As seen in Table 5, three beliefs emerged as significant unique predictors, explaining more than half of the variance (*R*^*2*^ = 51%) in intention to remove pubic hair. Feeling comfortable had the largest influence (*β* = .40), followed by feeling attractive (*β* = .35) and being painful (*β* = −.13), which was negatively associated with intention to engage in pubic hair removal.

**Table 4:** Means, standard deviations, and bivariate correlations for the underlying beliefs of intention to remove pubic hair

Belief	Mean	SD	r
*Behavioural Beliefs*			
Feeling attractive	5.48	1.57	.58**
Feeling clean	5.58	1.55	.55**
Being socially acceptable	4.74	1.75	.18**
Feeling comfortable	5.22	1.76	.65**
Reducing negative judgments from others	4.15	1.85	.15*
Being time consuming	5.67	1.48	−.15*
Being effortful	5.67	1.54	−.17**
Being expensive	3.96	2.00	−.16*
Being painful	4.27	1.98	−.24**
*Normative Beliefs*			
Friends	4.31	1.76	.18**
Partner	4.57	1.85	.34**
Media	5.79	1.35	− 0.08
Beauty industry	6.05	1.31	−0.11
Sex industry	6.05	1.34	−0.09
Alternative people/groups (e.g., hippies/naturalists)	2.67	1.37	0.12
Feminist groups	2.85	1.39	0.03
Society	5.59	1.41	−0.11
*Control beliefs*			
Lack of time	5.10	1.85	−.13*
Lack of equipment	3.75	2.03	−.22**
Laziness	5.58	1.67	−.18**

Note. * *p* < .05. ** *p* < .01

**Table 5:** Results of Stepwise Regression of Beliefs Underlying Intention to Remove Pubic Hair

	Belief	Β	SE	β	R^2^ (Adj. R^2^)	∆R^2^	sr^2^
Step 1	Feeling comfortable	.61	.05	.66***	.43 (.43)***	–	.43
Step 2	Feeling comfortable	.40	.06	.44***	.49 (.48)***	.06***	.17
	Feeling attractive	.33	.07	.33***			.10
Step 3	Feeling comfortable	.37	.06	.40***	.51 (.50)***	.02**	.15
	Feeling attractive	.35	.07	.35***			.12
	Being painful	−.11	.04	−.13**			.03

Note. Β = unstandardized coefficients; β = standardised coefficients; sr^2^ = squared semi-partial correlations. ** *p* < .01. *** *p* < .001

## Discussion

The current research aimed to expand understanding of the underlying decision-making processes leading young women to remove their pubic hair using established decision-making models. In line with the Theory of Planned Behaviour, it was hypothesised that attitudes, subjective norms, and perceived behavioural control would predict young women’s intention to engage in pubic hair removal (H1). Attitudes and perceived behavioural control emerged as significant predictors of intention to remove pubic hair in the next four weeks. It was further predicted that extending the standard Theory of Planned Behaviour by incorporating the Prototype Willingness Model constructs of prototype favourability and prototype similarity would add significantly to the prediction of intention to remove pubic hair (H2), as would the endorsement of feminist values (H3). Prototype similarity to pubic hair removers and non-removers emerged as significantly associated with intention to remove pubic hair. However, prototype favourability of removers and non-removers were not associated with intention. Feminist values was not significantly associated with intention to remove pubic hair but did emerge as a unique negative predictor of actual pubic hair removal. Finally, consistent with the Theory of Planned Behaviour, it was predicted that intention to engage in pubic hair removal and perceived behavioural control would predict self-reported pubic hair removal behaviour (H4). Intention was significantly positively associated with behaviour but perceived behavioural control was not. From the analyses of critical beliefs associated with intention to engage in removing pubic hair, three key beliefs emerged as significant. Beliefs that pubic hair removal makes women feel comfortable and attractive were positively associated with intention to remove pubic hair, while the belief that the hair removal process was painful was negatively associated.

### Utility of the theory of planned behaviour in understanding young Women’s pubic hair removal

In line with the theoretical assumptions of the Theory of Planned Behaviour, intention to remove pubic hair was significantly positively associated with self-reported pubic hair removal in the follow-up survey. Attitude and perceived behavioural control emerged as significant predictors of intention to remove pubic hair. Women who held positive attitudes and had greater confidence in their ability to remove their pubic hair had stronger intentions to engage in this behaviour. This finding aligns with the Theory of Planned Behaviour propositions and is unsurprising as women have been targeted by advertising selling the positive effects of hair removal and specific products to remove it [16]. Interestingly, subjective norm did not emerge as a unique predictor contrary to expectations based on previous literature. This finding indicates that peer and social pressure are less influential factors in the prevalence of women’s hair removal behaviours. As 98% of the sample reported that they had removed their pubic hair sometime in the past and over 50% reported currently having a Brazilian, it is possible that the pubic hair removal has become sufficiently normative that the young women in the study do not consider others’ belief as important in their consideration of pubic hair removal.

The findings from the addition of constructs of prototype similarity and favourability from the Prototype Willingness Model may add further explanation in the formation of young women’s intentions to remove their pubic hair. The significance of prototype similarity as a predictor of intention to remove pubic hair, but not prototype favourability, suggests that although young women did not consciously rate women who did or did not engage in pubic hair removal as explicitly more or less favourable, prototypes may have influenced young women’s intentions more subtly. The analyses of prototype traits showed that a prototypical pubic hair remover was rated as more “normal” than a non-remover. The most highly endorsed trait of a pubic hair remover was “clean”, while for a non-remover it was the positive trait “self-confident”. Viewing themselves as similar to the prototype of a women who removed their pubic hair, described as “normal” as well as with the positive trait “clean” did increase their intention to engage in the behaviour. In contrast, distancing themselves from women who did not remove their pubic hair, and so were not “normal” was also associated with intention to engage in pubic hair removal. Of interest is that the prototypical women who did not remove their pubic hair, were also endorsed as having positive traits such as self-confidence. This finding suggests that young women may hold a sense of admiration for non-removers as resisting social pressure to be “normal”. So, while social pressure is not at the forefront of the reasoned decision making in this context, there is still an awareness of its influence at some level.

The beliefs that emerged as most strongly linked with intention to remove pubic hair were that doing so makes you feel more attractive and more comfortable. The results are consistent with the findings of previous research, where these are commonly cited reasons for removing pubic hair (e.g., 7, 14) and attest to the messages young women are internalising regarding hair removal as clean and attractive.

### Influence of feminist values

Holding strong feminist values emerged as having a significant influence on young women’s pubic hair removal behaviour. While feminist values did not contribute significantly to the prediction of intention beyond attitudes, perceived behavioural control, and the prototype variables, this construct was a significant negative predictor of actual behaviour. Feminist ideology may be strongly connected to the notion of allowing women the autonomy to not remove their body hair; thus, this factor may play consciously on women’s actions rather than the formulation of intention per se.

### Practical implications

While hair removal is associated with a number of identified physical harms and complications, of more concern is young women’s psychological health and related body image issues associated with the social pressure for young women to engage in pubic hair removal. The findings from the current research may be useful in providing information for life skills and health education in schools, which aim to assist young women in making informed decisions about body image related behaviours including pubic hair removal practices. Questioning the positive attitudes young women hold of hair removal behaviour (by challenging the perceived advantages of attractiveness and comfort, and reinforcing the disadvantages these practices can cause, such as pain and discomfort) may prompt young women to query the accepted norms of this behaviour. Further, information that includes feminist perspectives may empower women and increase their autonomy in the decision-making regarding their own body.

### Strengths, limitations and future directions

The prototypes traits and underlying beliefs used in the current study were developed through the use of a pilot study. This piloting was key to understanding the prototypical images people hold of women that do or do not remove pubic hair. The implementation of the four-week follow-up survey allowed for the assessment of women’s actual behaviours to fully test the pathways of the Theory of Planned Behaviour. A key strength of this study is that it is the first to systematically assess women’s hair removal behaviours through the use of an established decision-making model such as the Theory of Planned Behaviour.

This study does have limitations that need to be noted when considering the findings. The participants for this study were self-selected and a majority of the sample were highly educated (61.6%), Caucasian (94.7%) women. There was also an unexpectedly high attrition rate between the main and the follow-up survey.

As the age of initiation of hair removal practices by women is becoming younger (13 years or less), future research should target the younger adolescent age group to better understand the developmental aspects of the internalising of social norms surrounding hair removal [31]. The current research was exploratory and there may be many other variables that influence young women’s decision to remove their pubic hair that are not considered here. For example, risk perceptions (Health Belief Model; 30), the interplay between barrier of pain of hair removal and desire to remove to increase perceived attractiveness, a deeper examination of the influence of partner’s beliefs, sexual identity and ethnicity may be an important constructs to consider within the context of the pubic hair removal practices. With changing social norms around pubic hair removal among men [6], exploring young males’ hair removal using a Theory of Planned Behaviour framework may also provide an interesting avenue for future research.

## Conclusions

The current study supported the theory of planned behaviour framework in understanding young women’s decision-making process regarding pubic hair removal. The addition of prototypes from the prototype willingness model framework demonstrated the influence these prototypes of a women who does or does not remove pubic hair can have on young women’s pubic hair removal behaviour. The findings of this study provide support for the use of these established decision-making models in exploring contemporary body image related behaviours such as pubic hair removal. The current study’s findings highlight the complex underpinnings associated with the social norm surrounding women’s pubic hair removal.

## Supplementary information

**Additional file 1.** Surveys.

## Abbreviations

PWM: Prototype Willingness Model
TPB: Theory of Planned Behaviour
