# Supplementary material for: A full Brazilian or all natural: understanding the influences on young women’s decision to remove their pubic hair
Source: BMC Womens Health. 2019 Dec 19;19:164. doi: 10.1186/s12905-019-0868-1 (PMC6921585; doi:10.1186/s12905-019-0868-1)
Supplement: Supplementary file 1 — Additional file 1. Surveys. [file 12905_2019_868_MOESM1_ESM.pdf]

Appendix A

Pilot Questionnaire

PARTICIPANT INFORMATION FOR QUT RESEARCH PROJECT
– Pilot –

An Exploration of Attitudes Underlying Young Women's Decision to Remove Public and Private Body Hair.

QUT Ethics Approval Number 1700000408

RESEARCH TEAM.

Principal Researcher: Ms Ebony Matthews Honours Student
Associate Researchers: Dr Patricia Obst Principal Supervisor
Professor Katherine White Associate Supervisor
School of Psychology and Counselling, Faculty of Health
Queensland University of Technology (QUT)

DESCRIPTION

This research project is being undertaken as part of an Honours thesis for Ebony Matthews.

The purpose of this research project is to examine the attitudes that influence hair removal practices among young women. Hair removal in this research will refer to both public hair removal (hair visible in clothes) and private hair removal (hair not usually visible in clothes).

You are invited to participate in this research project because you are a young woman aged between 17 and 25 years old living in Australia.

PARTICIPATION

Participation will involve completing an anonymous online survey that will take approximately 15 minutes of your time.

Questions will include:

- What areas of the body would you define as those involving public hair removal?
- Describe the typical woman who removes their private hair.
- What are the advantages of removing hair that is publically visible?

Your participation in this research project is entirely voluntary. If you agree to participate you do not have to complete any question(s) you are uncomfortable answering. Your decision to participate or not participate will in no way impact upon your current or future relationship with QUT (for example your grades). If you do agree to participate you can withdraw from the research project during

your participation without comment or penalty. However as the survey is anonymous, once it has been submitted it will not be possible to withdraw.

EXPECTED BENEFITS

It is expected that this research project will not directly benefit you. However, it may benefit the scientific and general community to better understand attitudes towards hair removal practices among young women and may be used to inform education campaigns.

RISKS

There are minimal risks associated with your participation in this research project. These include possible discomfort in response to questions about private hair removal practices, and personal beliefs and attitudes towards hair removal both publicly visible and privately visible. You can skip any questions that you wish to and you can opt out of the survey at any time.

QUT provides for limited free psychology, family therapy or counselling services (face-to-face only) for research participants of QUT projects who may experience discomfort or distress as a result of their participation in the research. Should you wish to access this service please call the Clinic Receptionist on **07 3138 0999** (Monday–Friday only 9am–5pm), QUT Psychology and Counselling Clinic, 44 Musk Avenue, Kelvin Grove, and indicate that you are a research participant.

Alternatively, Lifeline provides access to online, phone or face-to-face support, call **13 11 14** for 24 hour telephone crisis support. For young people aged under 25 years, you can also call the Kids Helpline on **1800 551 800**.

PRIVACY AND CONFIDENTIALITY

All comments and responses are anonymous and will be treated confidentially unless required by law. The names of individual persons are not required in any of the responses.

Any data collected as part of this research project will be stored securely as per QUT's Management of research data policy.

Please note that non-identifiable data from this p research roject may be used as comparative data in future research projects or stored on an open access database for secondary analysis.

CONSENT TO PARTICIPATE

Submitting the completed online survey is accepted as an indication of your consent to participate in this project.

QUESTIONS / FURTHER INFORMATION ABOUT THE RESEARCH PROJECT

If you have any questions or require further information please contact one of the listed researchers:

Ebony Matthews	ebony.matthews@connect.edu.au	
Patricia Obst	p.obst@qut.edu.au	07 3138 4931
Katherine White	km.white@qut.edu.au	07 3138 4689

CONCERNS / COMPLAINTS REGARDING THE CONDUCT OF THE RESEARCH PROJECT

QUT is committed to research integrity and the ethical conduct of research projects. However, if you do have any concerns or complaints about the ethical conduct of the research project you may contact the QUT Research Ethics Advisory Team on 07 3138 5123 or email humanethics@qut.edu.au. The QUT Research Ethics Advisory Team is not connected with the research project and can facilitate a resolution to your concern in an impartial manner.

THANK YOU FOR HELPING WITH THIS RESEARCH PROJECT.
PLEASE KEEP THIS SHEET FOR YOUR INFORMATION.

Introduction

Thank you for agreeing to participate in this research. You are here because you are a female aged between 17 and 25 years old and you live in Australia.

This survey is completely voluntary at all times and should you wish to withdraw, you may do so at anytime. All responses are non-identifiable. Email addresses and SONA codes are kept separately from responses at all times.

If this survey should bring up any difficult feelings please note that QUT provides for limited free psychology, family therapy or counselling services (face-to-face only) for research participants of QUT projects who may experience discomfort or distress as a result of their participation in the research and who live in Brisbane. Should you wish to access this service please call the Clinic Receptionist on 07 3138 0999 (Monday–Friday only, 9am–5pm), QUT Psychology and Counselling Clinic, 44 Musk Avenue, Kelvin Grove, and indicate that you are a research participant. Alternatively, Lifeline provides access to online, phone or face-to-face support, call 13 11 14 for 24-hour telephone crisis support. For young people aged between 5 and 25, you can also call the Kids Helpline on 1800 551 800.

If you have any further questions, please do not hesitate to contact the researchers:
Researcher: Ebony Matthews – ebony.matthews@connect.qut.edu.au
Principal Supervisor: Dr Patricia Obst – p.obst@qut.edu.au

Do you wish to participate in this survey?
Yes
No

1. What is your age?
16 or younger
17 - 25
26 or older

2. What is your sex?
Female
Male
Do not wish to specify

In your opinion, answer the following questions and statements.

3. What areas of the body would you consider as those involving the removal of hair that is publicly visible?

____
____
____

4. What areas of the body would you consider as those involving the removal of hair that is only privately visible?

____
_____
____
_____

Keeping in mind your previous responses of what public and private hair removal are, answer the following questions:

5. What do you see as the advantages of your engaging in the removal of hair that is publicly visible?

____
____
____

6. What do you see as the disadvantages of your engaging in the removal of hair that is publicly visible?

____
____
____

7. What do you see as the advantages of your engaging in the removal of hair that is only privately visible?

____
____
____

8. What do you see as the disadvantages of your engaging in the removal of hair that is privately visible?

____
____
____

When it comes to your engaging in hair removal, there might be individuals or groups who would think you should or should not perform this behaviour.

9. Please list the individuals or groups who would approve or think you should engage in the removal of hair that is publicly visible?

____
____
____

10. Please list the individuals or groups who would disapprove or think you should not engage in the removal of hair that is publicly visible?

____
____
____

11. Please list the individuals or groups who would approve or think you should engage in the removal of hair that is only privately visible?

____
____
____

12. Please list the individuals or groups who would disapprove or think you should not engage in the removal of hair that is only privately visible?

____
____
____

13. What factors or circumstances would help or encourage you to engage in the removal of hair that is publicly visible?

____
____
____

14. What factors or circumstances would prevent or discourage you from engaging in the removal of hair that is publicly visible?

____
____
____

15. What factors or circumstances would help or encourage you to engage in the removal of hair that is only privately visible?

____
____
____

16. What factors or circumstances would prevent or discourage you from engaging in the removal of hair that is only privately visible?

____
____

Think about what you imagine the type of person who engages in these different kinds of hair removal would be like.

19. What words can you think of that might describe a woman who engages in the removal of hair that is publicly visible.

20. What words can you think of that might describe a woman who does not engage in the removal of hair that is publicly visible.

21. What words can you think of that might describe a woman who engages in the removal of hair that is only privately visible.

22. What words can you think of that might describe a woman who does not engage in the removal of hair that is only privately visible.

23. Describe a scenario (situation, circumstance or event) that would prompt you to engage in the removal of hair that is publicly visible.

24. Describe a scenario (situation, circumstance or event) that would prompt you to engage in the removal of hair that is only privately visible.

THANK YOU FOR PARTICIPATING

Appendix B

Main Questionnaire

PARTICIPANT INFORMATION FOR QUT RESEARCH PROJECT
– Main Survey –

An Exploration of Attitudes Underlying Young Women's Decision to Remove Public and Private Body Hair.

QUT Ethics Approval Number 1700000408

RESEARCH TEAM.

Principal Researcher: Ms Ebony Matthews Honours Student
Associate Researchers: Dr Patricia Obst Principal Supervisor
Professor Katherine White Associate Supervisor
School of Psychology and Counselling, Faculty of Health
Queensland University of Technology (QUT)

DESCRIPTION

This research project is being undertaken as part of an Honours thesis for Ebony Matthews.

The purpose of this research project is to examine values (e.g. feminist values), beliefs, and attitudes (e.g. towards erotica/pornography) that may influence hair removal practices among young women. Hair removal in this research will refer to both public hair removal (hair visible in clothing) and private hair removal (hair not visible in clothing).

You are invited to participate in this research project because you are a young woman aged between 17 and 25 years old currently living in Australia.

PARTICIPATION

Participation will involve completing an anonymous online survey with mostly Likert scale answers (strongly agree – strongly disagree) that will take approximately 20-25 minutes of your time.

If you consent to be re-contacted via email (email addresses will be stored separately from your responses and will be deleted once sent), we will contact you 4 weeks later for a brief 5-10 minute follow-up survey. Survey responses will be matched via a unique code generated by you.

Examples of the types of questions in the survey include:
- Do you intend to remove your private hair in the next four weeks?
- Pornographic material increases the probability of sexual violence.

How vulnerable do you think you would be to getting a minor negative side effect (e.g. ingrown hairs, epidermal abrasions) from public hair removal in the next four weeks?

Your participation in this research project is entirely voluntary. If you agree to participate you do not have to complete any question(s) you are uncomfortable answering. Your decision to participate or not participate will in no way impact upon your current or future relationship with QUT (for example your grades). If you do agree to participate you can withdraw from the research project during your participation without comment or penalty. However as the survey is anonymous once it has been submitted it will not be possible to withdraw.

EXPECTED BENEFITS

It is not expected that this research project will directly benefit you. However, it may benefit the scientific and general community to better understand attitudes towards hair removal practices among young women and may be used to inform education campaigns.

To recognise your contribution should you choose to participate in this study (completing both this survey and a one month follow-up survey) the research team is offering eligible students enrolled in PYB100 and PYB102 at QUT .5% of course credit through the SONA system.

If you are not eligible for credit you are eligible to go into a draw to win one of two $50 Visa gift cards if you complete this survey and the one month follow-up survey. For those recruited through the SONA system you may choose either course credit or to go into the draw. Email addresses are required to go into the draw but these will be stored separately from survey responses and will be deleted once the prizes are claimed.

Please note the opening date for entries is 20/05/2017, the closing date for entries is 20/08/2017 and the prize/s are one of 2 $50 Visa gift cards.

The Terms and Conditions of the prize draw can be located at:
https://survey.qut.edu.au/survey-data/67/67667/media/62/6254.pdf

RISKS

There are minimal risks associated with your participation in this research project. These include possible discomfort in response to questions about private hair removal practices, and personal beliefs on private adult content (erotica). You can skip any questions you wish and you can opt out of the survey at any time.

QUT provides for limited free psychology, family therapy or counselling services (face-to-face only) for research participants of QUT projects who may experience discomfort or distress as a result of their participation in the research. Should you wish to access this service please call the Clinic Receptionist on **07 3138 0999** (Monday–Friday only 9am–5pm), QUT Psychology and Counselling Clinic, 44 Musk Avenue, Kelvin Grove, and indicate that you are a research participant.

Alternatively, Lifeline provides access to online, phone or face-to-face support, call **13 11 14** for 24 hour telephone crisis support. For young people aged under

25 years, you can also call the Kids Helpline on **1800 551 800**.

PRIVACY AND CONFIDENTIALITY

All comments and responses are anonymous and will be treated confidentially unless required by law. The names of individual persons are not required in any of the responses.

Any data collected as part of this research project will be stored securely as per QUT's Management of research data policy.
Please note that non-identifiable data from this research project may be used as comparative data in future research projects or stored on an open access database for secondary analysis.

CONSENT TO PARTICIPATE

Submitting the completed online survey is accepted as an indication of your consent to participate in this research project.

QUESTIONS / FURTHER INFORMATION ABOUT THE RESEARCH PROJECT

If you have any questions or require further information please contact one of the listed researchers:

Ebony Matthews	ebony.matthews@connect.edu.au	
Patricia Obst	p.obst@qut.edu.au	07 3138 4931
Katherine White	km.white@qut.edu.au	07 3138 4689

CONCERNS / COMPLAINTS REGARDING THE CONDUCT OF THE RESEARCH PROJECT

QUT is committed to research integrity and the ethical conduct of research projects. However, if you do have any concerns or complaints about the ethical conduct of the research project you may contact the QUT Research Ethics Advisory Team on 07 3138 5123 or email humanethics@qut.edu.au. The QUT Research Ethics Advisory Team is not connected with the research project and can facilitate a resolution to your concern in an impartial manner.

THANK YOU FOR HELPING WITH THIS RESEARCH PROJECT.
PLEASE KEEP THIS SHEET FOR YOUR INFORMATION.

Introduction

Thank you for agreeing to participate in this research. You are here because you are a female aged between 17 and 25 years old living in Australia.

This survey is completely voluntary at all times and should you wish to withdraw, you may do so at anytime. All responses are non-identifiable. Email addresses and SONA codes are kept separately from responses at all times.

If this survey should bring up any difficult feelings please note that QUT provides for limited free psychology, family therapy or counselling services (face-to-face only) for research participants of QUT projects who may experience discomfort or distress as a result of their participation in the research and who live in Brisbane. Should you wish to access this service please call the Clinic Receptionist on 07 3138 0999 (Monday–Friday only, 9am–5pm), QUT Psychology and Counselling Clinic, 44 Musk Avenue, Kelvin Grove, and indicate that you are a research participant. Alternatively, Lifeline provides access to online, phone or face-to-face support, call 13 11 14 for 24-hour telephone crisis support. For young people 25 years old or younger, you can also call the Kids Helpline on 1800 551 800.

If you have any further questions, please do not hesitate to contact the researchers:

Researcher: Ebony Matthews – ebony.matthews@connect.qut.edu.au
Principal Supervisor: Dr Patricia Obst – p.obst@qut.edu.au

Begin Survey

1. Do you wish to participate in this survey? Yes
No

2. What is your age? 16 or younger
17 - 25
26 or older

3. What is your sex? Female
Male
Do not wish to specify

UNIQUE IDENTIFIER CODE

TIME 1

Thank you very much for agreeing to participate in this survey.
All the information that you provide is anonymous and confidential.

ALL Participants please follow this example to create your Anonymous Code Identifier:

Example: Your first name is Michelle. Your mother's name is Jennifer. You were born on 17 June.

Your code identifier would be: MIJE17

M I	(a) What are the first 2 letters of your first name?
J E	(b) What are the first 2 letters of your mother's first name?
1 7	(c) What is the date (of the month) you were born on?

Your code identifier:

______ ______	(a) What are the first 2 letters of your first name?
______ ______	(b) What are the first 2 letters of your mother's first name?
______ ______	(c) What is the date (of the month) you were born on?

Demographic Questions

4. What ethnicity do you identify with?

Caucasian

Other ______

5. Which of the following do you identify with:

Homosexual

Bisexual

Heterosexual

Other ______

6. What is your relationship status?

Single

Casually dating

In a committed relationship

Married

Divorced

Widowed

7. What is your current employment status?

Employed full-time

Employed part-time

Employed casually

Unemployed

Permanently ill/unable to work

Other

8. What is your highest level of education completed or currently completing?

Primary School

High School

Vocational Certificate

Diploma or certificate taking 12 months or more full-time

Bachelor degree or higher

Postgraduate degree (masters or PhD)

9. Do you follow any cultural/religious practices that restrict the amount of skin publicly shown?

Yes

No

10. Before tax is taken out, which of the following ranges best describes your income, from all sources, over the last 12 months?

Less than $20,000

$20,001 - $30,000

$30,001 - $50,000

$50,001 - $100,000

$100,001 - $150,000

Over $150,000

Don’t know

Part B

Past Private Hair Removal Behaviour

The following questions will ask you about your past privately visible hair removal behaviour.

When answering the following questions, please keep the following definition of private hair removal behaviour in mind: Privately visible hair removal, is the removal of hair not visible in normal clothing (e.g. pubic hair, nipple hair, buttock hair).

33. Have you ever engaged in the removal of hair that was privately visible (e.g. pubic hair, nipple hair, buttock hair)?

Yes

No

34. In the last four weeks, did you engage in the removal of hair that was privately visible?

Yes

No

35. In the last four weeks, what was the most common method of removal you performed for hair that was privately visible?

Shaving

Waxing

Plucking

Removal creams

Laser hair removal/IPL

Electrolysis

36. In the last four weeks, how often did you engage in the removal of hair that was privately visible?

Never

Once

2 - 3 times

4 - 6 times

6 or more times

37. In the last four weeks, to what extent did you remove hair that was privately visible?

1<br>Not at all<br>☐	2<br>☐	3<br>☐	4<br>☐	5<br>☐	6<br>☐	7<br>A great extent<br>☐

38. In the last four weeks, how often did you remove hair that was privately visible?

1<br>Never<br>☐	2<br><br>☐	3<br><br>☐	4<br><br>☐	5<br><br>☐	6<br><br>☐	7<br>Very often<br>☐

39. Which of the following best describes your pubic hair status in the last four weeks?

Definitions:

Au-natural – no removal of pubic hair

Basic bikini – only pubic hair extending beyond the bikini line is removed

French/Playboy – only a narrow strip or small mound of pubic hair is left

Brazilian/Hollywood – full removal of all pubic hair from front to back

Au-natural

Basic bikini

French/Playboy

Brazilian/Hollywood

Attitudes and Perceived Vulnerability (privately visible)

The following questions will ask you about your current attitudes and beliefs towards the removal of hair that is privately visible.

For this question, please select the response that best indicates how you feel somewhere between each pair of words in response to the statement.

40. My engaging in the removal of hair that is privately visible, for the next four weeks would be:

Good	☐	☐	☐	☐	☐	☐	☐	Bad
Pleasant	☐	☐	☐	☐	☐	☐	☐	Unpleasant
Wise	☐	☐	☐	☐	☐	☐	☐	Foolish
Favourable	☐	☐	☐	☐	☐	☐	☐	Unfavourable
Right	☐	☐	☐	☐	☐	☐	☐	Wrong

41. How likely would it be that you would get a minor negative side effect (e.g. ingrown hairs, epidermal abrasions) from engaging in the removal of hair that is privately visible in the next four weeks?

Very unlikely	Unlikely	Somewhat Unlikely	Neither likely nor unlikely	Somewhat Likely	Likely	Very likely
☐	☐	☐	☐	☐	☐	☐

42. How likely would it be that you would get a major negative side effect (e.g. infections, sexually transmitted diseases) from engaging in the removal of hair that is privately visible in the next four weeks?

Very unlikely	Unlikely	Somewhat Unlikely	Neither likely nor unlikely	Somewhat Likely	Likely	Very likely
☐	☐	☐	☐	☐	☐	☐

Indirect Beliefs (privately visible)

43. How likely is it that your engaging in the removal of hair that is privately visible in the next four weeks will result in the following?:

	Very unlikel y	Unlikel y	Somewhat Unlikely	Neither likely nor unlikely	Somewhat Likely	Likel y	Very likely
Feel attractive	☐	☐	☐	☐	☐	☐	☐
Feel clean	☐	☐	☐	☐	☐	☐	☐
Be socially acceptable	☐	☐	☐	☐	☐	☐	☐
Feel comfortable	☐	☐	☐	☐	☐	☐	☐
Reduce negative judgments from others	☐	☐	☐	☐	☐	☐	☐
Be time consuming	☐	☐	☐	☐	☐	☐	☐
Be effortful	☐	☐	☐	☐	☐	☐	☐
Be expensive	☐	☐	☐	☐	☐	☐	☐
Be painful	☐	☐	☐	☐	☐	☐	☐

Subjective Norms (privately visible)

44. For engaging in the removal of hair that is privately visible in the next four weeks, do you agree that:

	Strongly Disagree	Disagree	Somewhat Disagree	Neither agree nor disagree	Somewhat Agree	Agree	Strongly Agree
Most people who are important to me would approve of my engaging in the removal of hair that is privately visible in the next four weeks.	1	2	3	4	5	6	7
Most people who are important to me think I should engage in the removal of hair that is privately visible in the next four weeks.	1	2	3	4	5	6	7
Most of my friends would engage in the removal of their own hair that is privately visible in the next four weeks.	1	2	3	4	5	6	7

Indirect Beliefs on Subjective Norms (privately visible)

45. How likely is it that the following people/groups would think you should engage in the removal of hair that is privately visible in the next four weeks?:

	Very unlikely	Unlikely	Somewhat unlikely	Neither likely nor unlikely	Somewhat Likely	Likely	Very likely	Not Applicable
Friends	1	2	3	4	5	6	7	☐
Partner	1	2	3	4	5	6	7	☐
Media	1	2	3	4	5	6	7	☐
Beauty industry	1	2	3	4	5	6	7	☐
Sex industry	1	2	3	4	5	6	7	☐
Feminist groups	1	2	3	4	5	6	7	☐
Alternative people/groups (e.g. hippies/ naturalists)	1	2	3	4	5	6	7	☐
Society	1	2	3	4	5	6	7	☐

Perceived Behavioural Control (privately visible)

46. For engaging in the removal of hair that is publicly visible in the next four weeks, do you agree that:

	Strongly Disagree	Disagree	Somewhat Disagree	Neither agree nor disagree	Somewhat Agree	Agree	Strongly Agree
I am confident that I can engage in the removal of hair that is privately visible in the next four weeks.	1	2	3	4	5	6	7
My engaging in the removal of hair that is privately visible in the next four weeks is up to me.	1	2	3	4	5	6	7

Indirect Beliefs on Behavioural Control (privately visible)

47. How likely in the next four weeks would the following factors stop you from engaging in the removal of hair that is privately visible:

	Very unlikely	Unlikely	Somewhat unlikely	Neither likely nor unlikely	Somewhat Likely	Likely	Very likely
Lack of time	1	2	3	4	5	6	7
Lack of equipment	1	2	3	4	5	6	7
Laziness	1	2	3	4	5	6	7

Prototypes (privately visible)

Now, we'd like you to think about the type of woman who engages in the removal of hair that is privately visible. We are not suggesting that all women who are like this are the same. However, we are interested in what traits you think this type of woman is likely to have.

Please pick one on each line.

48. How likely is this woman to be each of the following?:

	Very unlikely	Unlikely	Somewhat unlikely	Neither likely nor unlikely	Somewhat Likely	Likely	Very likely
Normal	1	2	3	4	5	6	7
Clean	1	2	3	4	5	6	7
Adventurous	1	2	3	4	5	6	7
Self-conscious	1	2	3	4	5	6	7

Now, we'd like you to think about the type of woman who does NOT engage in the removal of hair that is privately visible. We are not suggesting that all women who are like this are the same. However, we are interested in what traits you think this type of woman is likely to have.

Please pick one on each line.

49. How likely is this woman to be each of the following?:

	Very unlikely	Unlikely	Somewhat unlikely	Neither likely nor unlikely	Somewhat Likely	Likely	Very likely
Normal	1	2	3	4	5	6	7
Self-confident	1	2	3	4	5	6	7
Alternative/independent thinker (e.g. hippie)	1	2	3	4	5	6	7
Reserved	1	2	3	4	5	6	7
Unbothered by others' opinions	1	2	3	4	5	6	7

Prototype Favourability and Similarity (privately visible)

50. In general, how favourable is your impression of the type of woman who engages in the removal of hair that is privately visible?

Very Unfavourable	Unfavourable	Slightly unfavourable	Neither Favourable nor Unfavourable	Slightly Favourable	Favourable	Very Favourable
☐	☐	☐	☐	☐	☐	☐

51. In general, how favourable is your impression of the type of woman who does NOT engage in the removal of hair that is privately visible?

Very Unfavourable	Unfavourable	Slightly unfavourable	Neither Favourable nor Unfavourable	Slightly Favourable	Favourable	Very Favourable
☐	☐	☐	☐	☐	☐	☐

52. In general, how similar do you feel like to the type of woman who engages in the removal of hair that is privately visible?

Very Dissimilar	Dissimilar	Slightly Dissimilar	Neither Similar nor Dissimilar	Slightly Similar	Similar	Very Similar
☐	☐	☐	☐	☐	☐	☐

53. In general, how similar do you feel like to the type of woman who does NOT engage in the removal of hair that is privately visible?

Very Dissimilar	Dissimilar	Slightly Dissimilar	Neither Similar nor Dissimilar	Slightly Similar	Similar	Very Similar
☐	☐	☐	☐	☐	☐	☐

Behavioural Willingness (privately visible)

For the following scenarios please state how willing or not you would be to engage in the removal of hair that is publicly visible.

Scenario: You are going to the beach with some friends and are going to wear a bathing suit.

54. In this situation how willing would you be to:

	Very unwillin g	Unwillin g	Somewhat unwilling	Neither willing or unwilling	Somewhat willing	Willing	Very willing
Remove hair that is privately visible	☐	☐	☐	☐	☐	☐	☐
Not remove hair that is privately visible	☐	☐	☐	☐	☐	☐	☐

Scenario: You are going out on a date which you think will possibly lead to a sexual encounter that night.

55. In this situation how willing would you be to:

	Very unwillin g	Unwillin g	Somewhat unwilling	Neither willing or unwilling	Somewhat willing	Willing	Very willing
Remove hair that is privately visible	☐	☐	☐	☐	☐	☐	☐
Not remove hair that is privately visible	☐	☐	☐	☐	☐	☐	☐

Behavioural intention (privately visible)

	Strongly Disagree	Disagree	Somewhat Disagree	Neither agree nor disagree	Somewhat Agree	Agree	Strongly Agree
I intend to engage in the removal of hair that is privately visible in the next four weeks	1	2	3	4	5	6	7
It is likely that I will engage in the removal of hair that is privately visible in the next four weeks	1	2	3	4	5	6	7

Attitudes Towards Erotica

The following statements inquire about your thoughts and feelings towards erotica (pornography). For each item, indicate how well you agree on a scale from strongly disagree to strongly agree.

	Strongly Disagree	Disagre e	Neither agree nor disagree	Agre e	Strongly Agree
1. The availability of the material leads to a breakdown in community morals.	☐	☐	☐	☐	☐
2.This material should be made illegal	☐	☐	☐	☐	☐
3. I feel the material is offensive.	☐	☐	☐	☐	☐
4.<br>The material should be available to adults. (Reverse-coded)	☐	☐	☐	☐	☐
5. This material should be protected by the Australian Commission for Freedom of information, opinion, and expression. (Reverse-coded)	☐	☐	☐	☐	☐
6. The material increases the probability of sexual violence.	☐	☐	☐	☐	☐
7. The material exploits women.	☐	☐	☐	☐	☐
8. The material exploits men.	☐	☐	☐	☐	☐
9. In this material, the position and treatment of women are degrading to women.	☐	☐	☐	☐	☐
10. In this material, the position and treatment of men are degrading to men.	☐	☐	☐	☐	☐

Feminist Values

The following statements inquire about your thoughts and feelings towards gender discrimination and subordination (historical and current). For each item, indicate how well you agree on a scale from strongly disagree to strongly agree.

	Strongly Disagree	Disagre e	Slightly Disagree	Agree Slightly	Agree	Strongly Agree
1. Even though some things have changed, women are still treated unfairly in today's society.	☐	☐	☐	☐	☐	☐
2. Women have been treated unfairly on the basis of their gender throughout most of human history.	☐	☐	☐	☐	☐	☐
3.The achievements of women in history have not been emphasized as much as those of men.	☐	☐	☐	☐	☐	☐
4. Men have too much influence in Australian politics compared to women.	☐	☐	☐	☐	☐	☐
5. People who complain that pornography treats women like objects are overreacting.(Reverse)	☐	☐	☐	☐	☐	☐
6. Men still don't take women's ideas seriously.	☐	☐	☐	☐	☐	☐
7. Women are already given equal opportunities with men in all important sectors of their lives. (Reverse)	☐	☐	☐	☐	☐	☐
8. Women have fewer choices available to them as compared to men.	☐	☐	☐	☐	☐	☐
9. Women in Australia are treated as second-class citizens.	☐	☐	☐	☐	☐	☐
10. All men receive economic, sexual, and psychological benefits from male domination.	☐	☐	☐	☐	☐	☐

Appendix C

Follow-Up Questionnaire (Time 2)

?	**PARTICIPANT INFORMATION FOR QUT RESEARCH PROJECT**<br>**– Follow-up Survey –**

An Exploration of Attitudes Underlying Young Women's Decision to Remove Public and Private Body Hair.

QUT Ethics Approval Number 1700000408

RESEARCH TEAM.

Principal Researcher: Ms Ebony Matthews Honours Student
Associate Researchers: Dr Patricia Obst Principal Supervisor
Professor Katherine White Associate Supervisor
School of Psychology and Counselling, Faculty of Health
Queensland University of Technology (QUT)

DESCRIPTION

This research project is being undertaken as part of an Honours thesis for Ebony Matthews.

The purpose of this research project is to examine body hair removal practices among young women.

You must have previously filled in the initial survey four weeks ago to participate in the following survey.

You are invited to participate in this research project because you are a young woman aged between 17 and 25 years old and currently live in Australia.

PARTICIPATION

Participation will involve completing an anonymous online survey that will take approximately 5-10 minutes of your time.

Questions will include:
In the last four weeks, did you remove your private body hair?
What was the most common method of private hair removal you performed in the last four weeks?

Your participation in this research project is entirely voluntary. If you agree to participate you do not have to complete any question(s) you are uncomfortable answering. Your decision to participate or not participate will in no way impact upon your current or future relationship with QUT (for example your grades). If

you do agree to participate you can withdraw from the research project during your participation without comment or penalty. However as the survey is anonymous once it has been submitted it will not be possible to withdraw.

EXPECTED BENEFITS

It is expected that this research project will not directly benefit you. However, it may benefit the scientific and general community to better understand attitudes towards hair removal practices among young women and may be used to inform education campaigns.

To recognise your contribution should you choose to participate in this follow-up survey (having completed the first survey) the research team is offering eligible students enrolled in PYB100 and PYB102 at QUT .5% of course credit through the SONA system.

If you are not eligible for credit you are eligible to go into a draw to win one of two $50 Visa gift cards if both the survey and follow-up survey have been completed. For those recruited through the SONA system you may choose either course credit or to go into the draw. Email addresses are required to go into the draw but these will be stored separately from survey responses and will be deleted once the prizes are claimed.

Please note the opening date for entries is 20/05/2017, the closing date for entries is 20/08/2017 and the prize/s are one of 2 $50 Visa gift cards.

The Terms and Conditions of the prize draw can be located at:
https://survey.qut.edu.au/survey-data/67/67667/media/62/6254.pdf

RISKS

There are minimal risks associated with your participation in this research project. These include possible discomfort in response to questions about private hair removal behaviours. You can skip any questions you wish and you can opt out of the survey at any time.

QUT provides for limited free psychology, family therapy or counselling services (face-to-face only) for research participants of QUT projects who may experience discomfort or distress as a result of their participation in the research. Should you wish to access this service please call the Clinic Receptionist on **07 3138 0999** (Monday–Friday only 9am–5pm), QUT Psychology and Counselling Clinic, 44 Musk Avenue, Kelvin Grove, and indicate that you are a research participant.

Alternatively, Lifeline provides access to online, phone or face-to-face support, call **13 11 14** for 24 hour telephone crisis support. For young people aged under 25 years, you can also call the Kids Helpline on **1800 551 800**.

PRIVACY AND CONFIDENTIALITY

All comments and responses are anonymous and will be treated confidentially unless required by law. The names of individual persons are not required in any of the responses.

Any data collected as part of this research project will be stored securely as per QUT's Management of research data policy.

Please note that non-identifiable data from this research project may be used as comparative data in future research projects or stored on an open access database for secondary analysis.

CONSENT TO PARTICIPATE

Submitting the completed online survey is accepted as an indication of your consent to participate in this research project.

QUESTIONS / FURTHER INFORMATION ABOUT THE RESEARCH PROJECT

If you have any questions or require further information please contact one of the listed researchers:

Ebony Matthews	ebony.matthews@connect.edu.au	
Patricia Obst	p.obst@qut.edu.au	07 3138 4931
Katherine White	km.white@qut.edu.au	07 3138 4689

CONCERNS / COMPLAINTS REGARDING THE CONDUCT OF THE RESEARCH PROJECT

QUT is committed to research integrity and the ethical conduct of research projects. However, if you do have any concerns or complaints about the ethical conduct of the research project you may contact the QUT Research Ethics Advisory Team on 07 3138 5123 or email humanethics@qut.edu.au. The QUT Research Ethics Advisory Team is not connected with the research project and can facilitate a resolution to your concern in an impartial manner.

THANK YOU FOR HELPING WITH THIS RESEARCH PROJECT.
PLEASE KEEP THIS SHEET FOR YOUR INFORMATION.

Introduction

Thank you for agreeing to participate in this research. You are here because you are a female aged between **17 and 25 years old** who completed a survey four weeks previously.
This survey is completely voluntary at all times and should you wish to withdraw, you may do so at anytime. All responses are non-identifiable. Email addresses and SONA codes are kept separately from responses at all times.

If this survey should bring up any difficult feelings please note that QUT provides for limited free psychology, family therapy or counselling services (face-to-face only) for research participants of QUT projects who may experience discomfort or distress as a result of their participation in the research and who live in Brisbane. Should you wish to access this service please call the Clinic Receptionist on 07 3138 0999 (Monday–Friday only, 9am–5pm), QUT Psychology and Counselling Clinic, 44 Musk Avenue, Kelvin Grove, and indicate that you

are a research participant. Alternatively, Lifeline provides access to online, phone or face-to-face support, call 13 11 14 for 24-hour telephone crisis support. For young people 25 years old or younger, you can also call the Kids Helpline on 1800 551 800.

If you have any further questions, please do not hesitate to contact the researchers:

Researcher: Ebony Matthews – ebony.matthews@connect.qut.edu.au

Principal Supervisor: Dr Patricia Obst – p.obst@qut.edu.au

Begin Survey

1. Do you wish to participate in this survey?
Yes
No

2. Did you previously complete the main study on hair removal?
Yes
No

UNIQUE IDENTIFIER CODE

TIME 1

Thank you very much for agreeing to participate in this survey.
All the information that you provide is anonymous and confidential.

ALL Participants please follow this example to create your Anonymous Code Identifier:

Example: Your first name is Michelle. Your mother's name is Jennifer. You were born on 17 June.

Your code identifier would be: MIJE17

M I	(a) What are the first 2 letters of your first name?
J E	(b) What are the first 2 letters of your mother's first name?
1 7	(c) What is the date (of the month) you were born on?

Your code identifier:

______ ______	(a) What are the first 2 letters of your first name?
______ ______	(b) What are the first 2 letters of your mother's first name?
______ ______	(c) What is the date (of the month) you were born on?

Past Private Hair Removal Behaviour

The following questions will ask you about your past privately visible hair removal behaviour.

When answering the following questions, please keep the following definition of private hair removal behaviour in mind: Privately visible hair removal, is the removal of hair not visible in normal clothing (e.g. pubic hair, nipple hair, buttock hair).

3. Have you ever engaged in the removal of hair that was privately visible (e.g. pubic hair, nipple hair, buttock hair)?

Yes

No

4. In the last four weeks, did you engage in the removal of hair that was privately visible?

Yes

No

5. In the last four weeks, what was the most common method of removal you performed for hair that was privately visible?

Shaving

Waxing

Plucking

Removal creams

Laser hair removal/IPL

Electrolysis

6. In the last four weeks, how often did you engage in the removal of hair that was privately visible?

Never

Once

2 - 3 times

4 - 6 times

6 or more times

7. In the last four weeks, to what extent did you remove hair that was privately visible?

1<br>Not at all<br>☐	2<br><br>☐	3<br><br>☐	4<br><br>☐	5<br><br>☐	6<br><br>☐	7<br>A great extent<br>☐

8. In the last four weeks, how often did you remove hair that was privately visible?

1<br>Never<br>☐	2<br><br>☐	3<br><br>☐	4<br><br>☐	5<br><br>☐	6<br><br>☐	7<br>Very often<br>☐

15. Which of the following best describes your pubic hair status in the last four weeks?

Definitions:

Au-natural – no removal of pubic hair

Basic bikini – only pubic hair extending beyond the bikini line is removed

French/Playboy – only a narrow strip or small mound of pubic hair is left

Brazilian/Hollywood – full removal of all pubic hair from front to back

Au-natural

Basic bikini

French/Playboy

Brazilian/Hollywood

THANK YOU FOR PARTICIPATING!

Please click the link to enter your email address to go into the running to win one of two $50 VISA gift-vouchers.

For SONA eligible participants, please click the SONA link to enter your 5-digit SONA code for your 1% course credit for participating in this research.

THE LINKS WILL OPEN IN NEW WINDOWS AND AT NO TIME CAN BE LINKED TO YOUR RESPONSES.

PRIZE DRAW LINK HERE

SONA LINK HERE
